# Early Presentation of an Ulcerated Infantile Haemangioma in a Newborn

**DOI:** 10.7759/cureus.21545

**Published:** 2022-01-24

**Authors:** Filipa Carvalho, Maria Liberal, Filipa Vale, Nuno Rodrigues Santos, Rui Guedes

**Affiliations:** 1 Family Medicine, ACeS Ave - Famalicão, USF S. Miguel o Anjo, Vila Nova de Famalicão, PRT; 2 Family Medicine, ACeS Ave Famalicão, USF S.Miguel o Anjo, Vila Nova de Famalicão, PRT; 3 Family Medicine, ACeS Ave-Famalicão, USF S. Miguel o Anjo, Vila Nova de Famalicão, PRT; 4 Neonatology, Centro Hospitalar Universitário de São João, Porto, PRT; 5 Paediatrics, Centro Hospitalar Universitário de São João, Porto, PRT

**Keywords:** infantile haemangioma, propanolol, wound care treatment, ulceration, newborn

## Abstract

Infantile haemangiomas (IHs) are the most common benign soft tissue tumours in children. Usually, they evolve without clinical incurrences and regression of the lesion can occur spontaneously in the first years of life. The decision for treatment is dependent upon the intrinsic characteristics of the lesion such as location, extension, functional compromise and complications.

We present the case of a newborn who was clinically accessed during ambulatory routine consultation when a lesion with 5x5 centimetres compatible with an IH was first observed. Inflammatory signs with no active bleeding were present and the newborn displayed signs of discomfort during a diaper change and manipulation of the anogenital area. For this reason, a referral was made for observation in a central hospital with specialised paediatrics, paediatric surgery and dermatology support. A 10-day antibiotic course with flucloxacillin and local topical care with silver sulfadiazine cream and barrier cream with zinc oxide were adopted, achieving a good clinical outcome. Laboratory workup, cardiovascular assessment, imagiological investigation with abdominopelvic and spinal cord ultrasonography as well as lumbosacral magnetic resonance imaging were all normal.

Ulceration is the most prevalent complication of IHs and it is associated with pain, recurrent bleeding, infection and difficult scarring, thus early recognition and directed treatment are essential to achieve a good clinical outcome.

## Introduction

Infantile haemangiomas (IHs) are the most common non-malignant vascular type of tumours in paediatric age affecting up to 5%-10% of infants in the first years of life [1,2]. The underlying etiopathogenetic factors for IHs formation are not yet fully understood. However, it is observed in clinical practice and international reviews also describe a predilection for their occurrence in female Caucasians. Other risk factors were proposed such as low birth weight, prematurity, advanced maternal age, multiple gestations, in-vitro fertilisation, pre-eclampsia, and placental malformations [1-3].

The natural history of IHs comprises a biphasic cycle with a proliferative and an involution phase. The proliferation period typically starts in the first weeks of life and continues up to the 6-12 months of age and it is characterised by rapid growth of the lesion. The involution phase takes place afterwards and it is determined by the cessation of the lesion’s growth with stabilisation in its size, followed by a progressive resolution of the initial vascular lesion in the following years, with or without direct intervention [1,2,4].

Partial or complete resolution of IHs is observed in 80%-90% of the cases in the first decade of life without any functional or cosmetic sequelae [1,2,4]. However, after the involution phase occurs, residual scarring, telangiectasia or local skin atrophy can occur in some cases and complete resolution appears to be dependent on the early onset of their involution phase [3]. Even though most IHs evolve in a benign manner, a small subgroup of patients can be at risk for complications and poor clinical outcomes if early identification and intervention do not occur [5,6].

IHs classification is therefore essential for risk stratification and assisting clinical decisions for conservative or interventional management [5,6]. IHs were classically classified based on the extent of soft tissue involvement as superficial, deep or mixed haemangiomas. However, morphologic subtype classification of IHs as segmental, indeterminate, focal/multifocal appears to provide more relevant clinical data as the greatest predictor of complications. Focal IHs are single lesions that comprise a single focal point and are the most common type, while multifocal IHs affect more than one different cutaneous area. Both subtypes are less prone to complications. Segmental IHs typically involve a large area of skin in a plaque-like manner displaying a pattern suggestive of an underlying embryonal development defect. These are the least common subtype but the most prone to complications. Those who have mixed features and cannot be clearly defined as focal or segmental are classified as indeterminate [5,6].

The diagnosis of IHs is clinical and is based on the history of the lesion’s evolution and clinical examination. However, imaging may be needed to reach a diagnosis in certain cases [1-6]. As previously mentioned, most uncomplicated IHs evolve in a benign way with no need for direct intervention; therefore, conservative management with ambulatory follow-up of the lesion is the most common strategy employed. Nevertheless, intrinsic patient factors (such as gender, ethnicity, low birth weight), the evolutionary phase of the IH, lesion’s size and anatomical location as well as the morphologic subtype of the IH, can determine the need for a stricter vigilance, requiring direct pharmacological or local intervention. This might especially occur in the 10% of cases when there is a direct life threat, involvement of vital organs, functional compromise, recurrent ulceration of the lesion and unacceptable aesthetic compromise [4-6].

Since the accidental discovery in 2008 of the beneficial effect of propranolol in promoting IHs’ regression, beta-blockers have become the mainstay of therapy with good efficacy and a relatively low-risk profile, substituting the widely used corticosteroids (intralesional and systemic) as the main pharmacological strategy [3,4,5,7]. Other pharmacological modalities previously described in the literature included imiquimod, vincristine, bleomycin A5, and alfa-interferon [3,4,5,7]. Surgery and laser therapy are other local interventions that can be used in some selected cases [3,4,5,7].

## Case presentation

**Figure 1:**
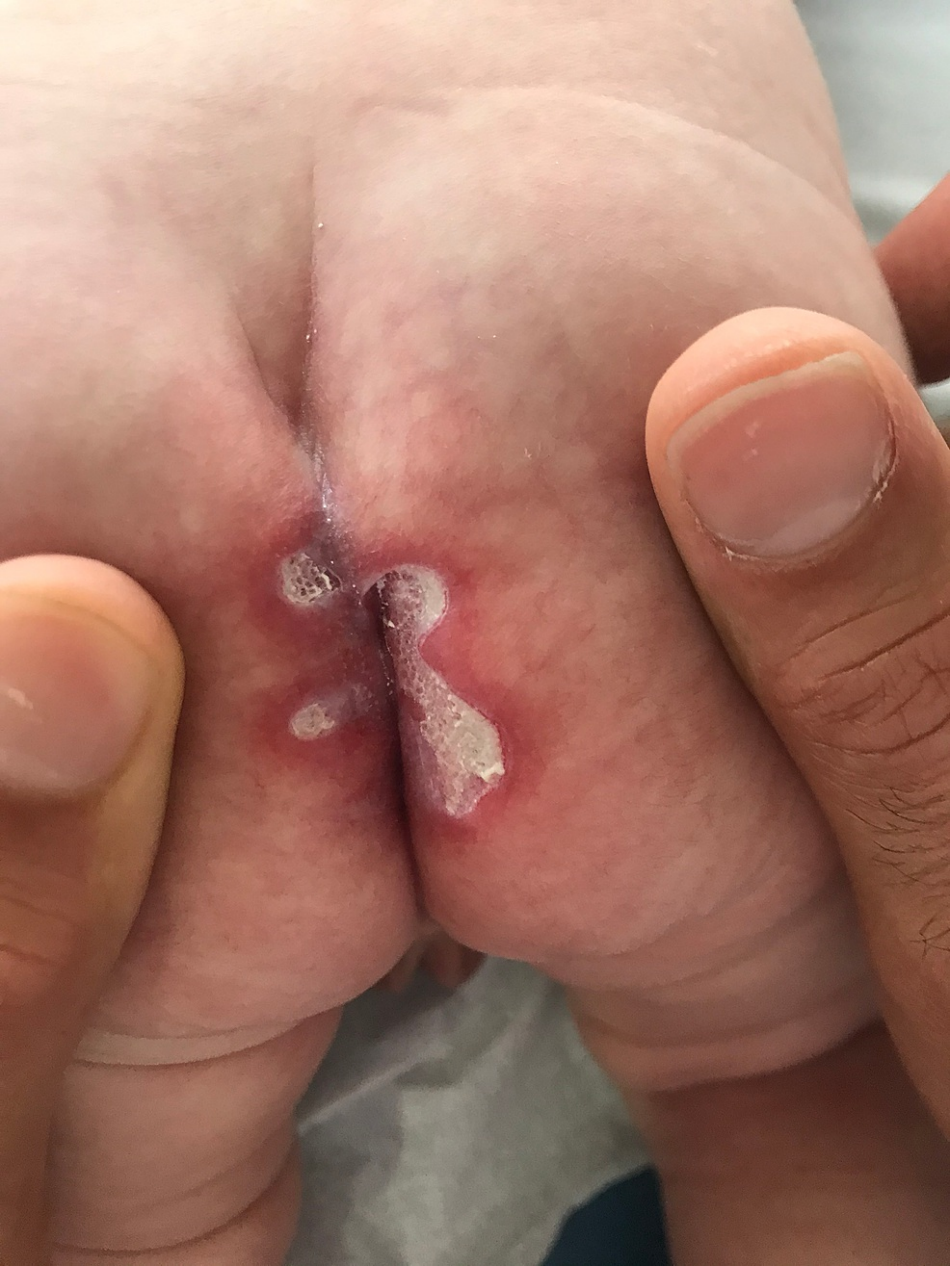
Red lesion in the intergluteal cleft with intense erythema and areas of fibrinous deposits and white exudate.

**Figure 2:**
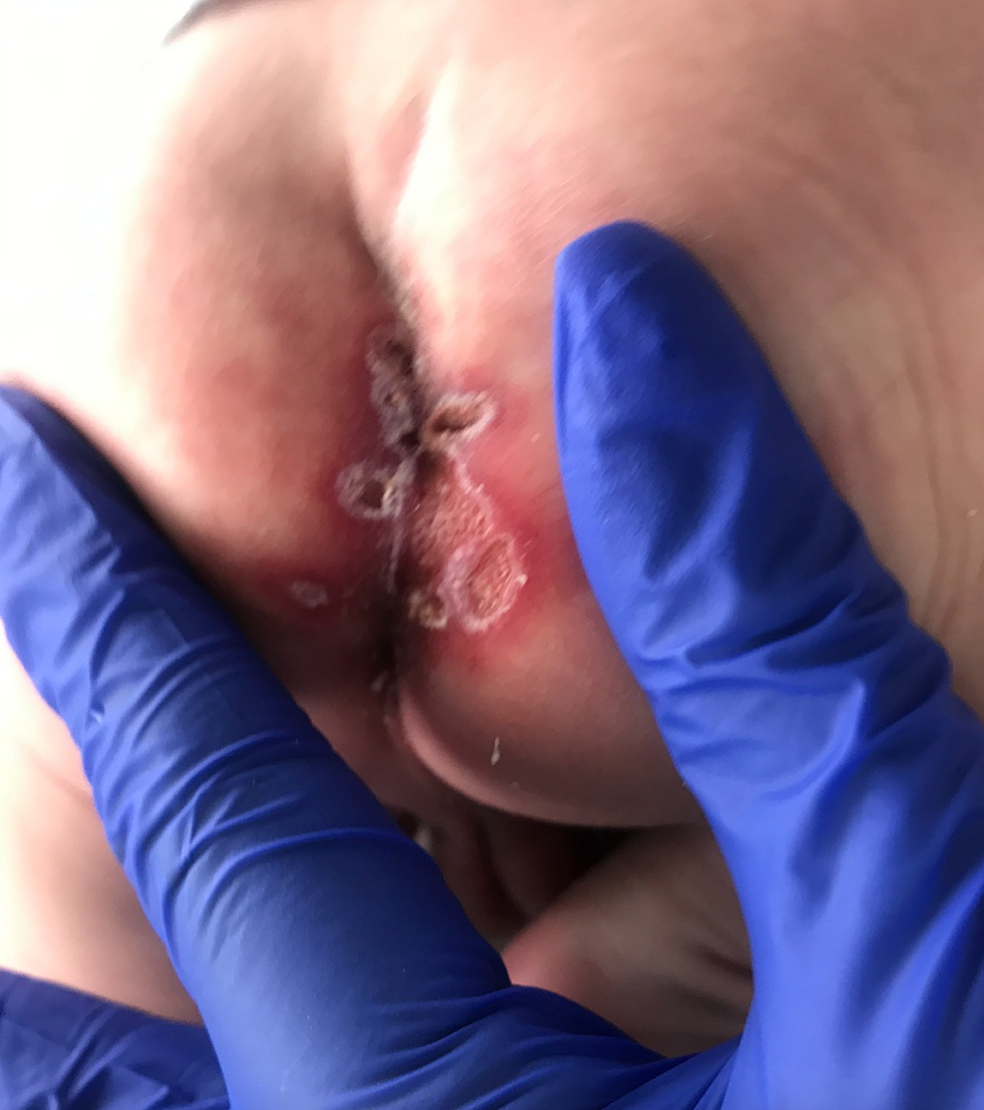
Red lesion in the intergluteal cleft with flat ulcers.

We present the case of a 17-day-old term male newborn with non-contributory prenatal and family history, who was delivered in a central hospital with a normal adaptation to an extra-uterine life environment. Hospital in-stay physical examinations were unremarkable and he was clinically discharged after the third day postpartum without any clinical incurrences. Approximately around eight days of life, the newborn’s mother took notice of a red lesion in the intergluteal cleft which was not present at birth. During the following weeks, she applied protective barrier cream in the lesion three times a day, but the lesion’s area and its associated erythema progressively increased. For this reason, when he was 17 days old the mother brought him to an ambulatory routine consultation where the lesion was first observed. During the examination, the newborn displayed normal vitals and good interaction with the physician but upon manipulation of the diaper and the intergluteal cleft area, he appeared irritated and uncomfortable. At this point, a 5x5 centimetres red lesion with areas of intense erythema and flat ulcers with fibrinous deposits and some exudate was present (Figures 1, 2).

The newborn was then referred for observation and follow-up in a central hospital with differentiated support from paediatrics, paediatric surgery and dermatology. Initial laboratory workup at admission did not show elevation of any inflammatory markers. Upon clinical suspicion of a vascular tumour as the underlying cause for the ulcerated lesion, tumour markers were performed and elevation in alfa-fetoprotein levels was present. Initial laboratory workup and culture results are presented in Table 1. Imagiological study with abdominopelvic and spinal cord ultrasonography, followed up by lumbosacral magnetic resonance imaging (MRI), were performed and no occult spinal dysraphism, masses or vascular anomalies were found.

**Table 1:** Summary of laboratory workup upon hospital admission.

Analytical Workup	
Haemoglobin	12.8 g/dL
Leucocytes	11,000 U/L (Neutrophiles 3,600 U/L [32.8%])
Platelets	451,000 U/L
Glucose	93 mg/dL
Sodium	140 mmol/L
Potassium	5.6 mmol/L
Chlorine	107 mmol/L
Urea	11 mg/dL
Creatinine	0.2 mg/dL
C-Reactive Protein	< 0.5 mg/L
Procalcitonin	0.08 ng/mL
Beta-Human Chorionic Gonadotropin	<2 mUI/mL
Alfa-fetoprotein	3,327.14 ng/mL
Haemocultures	Negative
Lesion’s Exudate Bacteriologic Culture	Polymicrobial (Enterococcus faecalis, Escherichia coli, Klebsiella pneumoniae spp, Morganella morgani, Streptococcus lutetiensis, Citrobacter freundii, Enterococcus faecalis, Anaerococcus spp)

**Figure 3:**
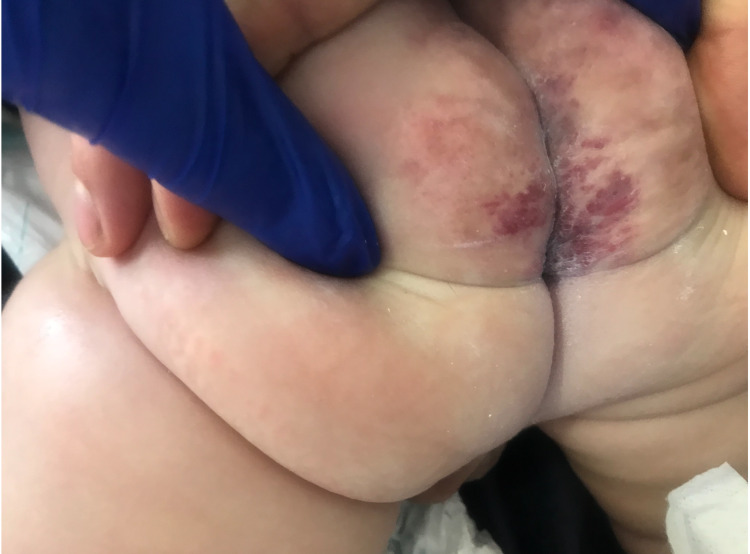
Clinical aspect and scarring process of the lesion two months after the initial episode during ambulatory follow-up.

**Figure 4:**
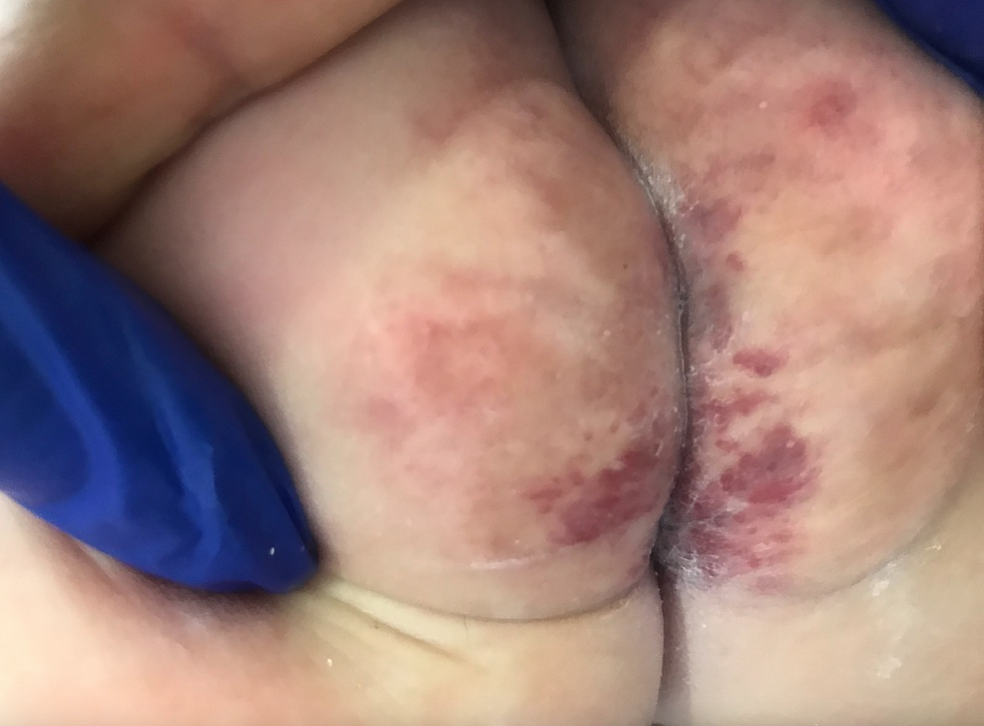
Scarred infantile haemangioma in the intergluteal cleft after two months of treatment.

The perianal lesion presented with a good clinical evolution with adequate scarring and regression of the inflammatory signs after 10 days of treatment. The newborn was then discharged with the likely diagnosis of an ulcerated IH and was referred to paediatric surgery consultation for the lesion’s ambulatory follow-up. A paediatric cardiology assessment was performed when the infant was three months old. His electrocardiogram and echocardiogram were normal therefore no contraindications for beta-blocker treatment (if needed) were present. He is currently four months old and during the follow-up period, no other ulcerative lesions or dimensional progression of the lesion occurred (Figures 3, 4). Topical care with daily application of barrier cream during diaper changes is being performed. Despite a good clinical outcome, he remains under strict follow-up and other therapeutical approaches may be needed depending on the lesion’s evolution in the following months.

## Discussion

The clinical presentation and evolution of IHs can make the diagnosis very challenging, especially when the primary lesion is associated with further complications when it is first observed [1]. Therefore, physicians’ awareness of IHs’ presentation and their complications can facilitate an early diagnostic recognition, allowing timely wise intervention. Ulceration is the most common complication of IHs with an estimated incidence of 15% [1,8,9,10]. Intrinsic aspects of the lesion can make them more prone to ulceration, such as superficial and mixed haemangiomas, larger lesions and those located in intertriginous areas exposed to friction. Following the process of ulceration, other problems can arise such as secondary infection, difficult pain management and functional compromise [10]. Anatomical aspects of the region where the ulcerated IHs are located are also important. Anogenital ulcerated IHs can be particularly hard to treat because of secondary contact with urine and faeces in the earlier stages of infancy. IHs typically tend to develop ulcerations during the proliferative phase and macroscopically the early white discoloration of the lesion is recognised as a predictive clinical sign of future ulceration [10].

The management of ulcerated IHs is constantly evolving and different approaches can be held. Currently, initial local wound care, pharmacologic therapy, laser or surgical excision are the main options for treatment and frequently different methods are used concomitantly [5,7,9].

According to Wang et al.'s systematic review of ulcerated IHs’ management involving 78 studies and a total of 1230 patients with pediatric age, wound care with atraumatic and non-adhesive dressing was the most commonly agreed-on management strategy, with antibiotics being reserved for concomitant infection of the wound. Propranolol was the most used systemic agent in a total of 28 studies, with dosages ranging from 1.5 to 3.0 mg/kg/d. With this therapeutic regimen, 97% of wounds healed with a mean healing time of 5.7 weeks [9]. As a beta-blocker alternative, timolol 0.5% was preferentially used for the approach of smaller haemangiomas with smaller areas of ulceration when the side effects of systemic therapy were not negligible [8,9]. In eight studies with a total of 225 patients, pulsed dye laser was used with a rate of 100% of wound healing and shorter mean times for healing compared to propranolol [9]. Surgical resections were typically performed for larger haemangiomas and those causing complications such as suffocation, haemorrhage or disfigurement because in these cases it was dangerous to wait for medical therapy to be effective in the meantime [9].

In our clinical case, the newborn displayed a single 5x5 centimetres lesion which became apparent in the first week of life. As predictive risk factors, he was Caucasian and the lesion was localised in the intergluteal cleft, which is an area highly prone to friction. Despite the lesion’s dimension the newborn only displayed discomfort with direct manipulation of the lesion and/or the diaper. However, he was only 17 days old and with such lesion there was a high risk of subsequent infection and sepsis, therefore he had to be admitted to the hospital so that further potential complications could be safely excluded and urgent intravenous medication could be prescribed.

No signs of other occult haemangiomas or vascular malformations and masses were found throughout the imagiological study. This fact led the medical team to adopt a conservative strategy with a systemic antibiotic course, strict wound care supervision and frequent dressing revision, while silver sulfadiazine cream and another topical barrier cream with zinc were being applied. During the 10-day antibiotic course, the lesion showed excellent scarring and the newborn did not show signs of discomfort, pain, irritation, poor feeding periods or development compromise.

The presented case report highlights the challenge involved in the treatment of complicated IHs. Treatment options are diverse, but they should always be adapted upon the patient’s medical background, the characteristics of the lesion, feasibility, cost and parental agreement with the modality chosen for wound treatment.

## Conclusions

With the presented case report the authors would like to emphasise the clinical challenges involved with the diagnosis and management of a complicated IH in a newborn. In this case, a prompt diagnosis and intervention were mandatory to achieve a better clinical outcome and avoid other associated complications such as secondary infection and recurrent haemorrhage, compromising the newborn’s normal development and wellbeing.

The anogenital location demanded a multidisciplinary approach to exclude a possible syndromic manifestation where spinal cord dysraphism and anogenital anomalies could also be present. Only after proper exclusion of other organ or vascular structures compromise the treatment course and follow-up were defined. Despite the increasing use of beta-blockers as the mainstay for complicated IHs who require direct intervention, in our case, a conservative approach was deemed acceptable after multidisciplinary evaluation, with a good clinical outcome being achieved. Nevertheless, future intervention with directed pharmacological therapy remains possible depending on the lesion’s natural evolution in the future.
